# Transmission pattern of shigellosis in Wuhan City, China: a modelling study

**DOI:** 10.1017/S0950268821002363

**Published:** 2021-11-02

**Authors:** Zeyu Zhao, Qi Chen, Bin Zhao, Qingqing Hu, Jia Rui, Yao Wang, Yuanzhao Zhu, Xingchun Liu, Jingwen Xu, Meng Yang, Meijie Chu, Yanhua Su, Benhua Zhao, Tianmu Chen

**Affiliations:** 1 State Key Laboratory of Molecular Vaccinology and Molecular Diagnostics, School of Public Health, Xiamen University, Fujian, People's Republic of China; 2 Hubei Provincial Center for Disease Control and Prevention, Wuhan City, Hubei Province, People's Republic of China; 3 Laboratory Department, Xiang'an Hospital of Xiamen University, State Key Laboratory of Molecular Vaccinology and Molecular Diagnostics, Xiamen, Fujian, People's Republic of China; 4 Division of Public Health, School of Medicine, University of Utah, 201 Presidents Circle, Salt Lake City 84112, Utah, USA

**Keywords:** Effective reproduction number, mathematical model, meteorological factors, shigellosis, transmissibility

## Abstract

The article aims to estimate and forecast the transmissibility of shigellosis and explore the association of meteorological factors with shigellosis. The mathematical model named Susceptible–Exposed–Symptomatic/Asymptomatic–Recovered–Water/Food (SEIARW) was used to explore the feature of shigellosis transmission based on the data of Wuhan City, China, from 2005 to 2017. The study applied effective reproduction number (*R*_eff_) to estimate the transmissibility. Daily meteorological data from 2008 to 2017 were used to determine Spearman's correlation with reported new cases and *R*_eff_. The SEIARW model fit the data well (*χ*^2^ = 0.00046, *p* > 0.999). The simulation results showed that the reservoir-to-person transmission of the shigellosis route has been interrupted. The *R*_eff_ would be reduced to a transmission threshold of 1.00 (95% confidence interval (CI) 0.82–1.19) in 2035. Reducing the infectious period to 11.25 days would also decrease the value of *R*_eff_ to 0.99. There was a significant correlation between new cases of shigellosis and atmospheric pressure, temperature, wind speed and sun hours per day. The correlation coefficients, although statistically significant, were very low (<0.3). In Wuhan, China, the main transmission pattern of shigellosis is person-to-person. Meteorological factors, especially daily atmospheric pressure and temperature, may influence the epidemic of shigellosis.

## Introduction

In 1892, dysentery was described as ‘one of the four great epidemic diseases of the world’ by the eminent physician William Osler [1]. Shigellosis (or bacterial dysentery) caused by *shigella* is an acute intestinal infectious disease that often occurs in summer and autumn. High-risk groups in high-income countries include travellers and MSM (men who have sex with men), and in low- and middle-income countries include children aged 1–4 years [1]. *Shigella* spp. are among the causes of diarrhoea in children [3]. Despite reductions in morbidity and mortality of shigellosis in the past 30 years, shigellosis led to approximately 164 000 annual deaths worldwide [4, 5].

With China's rapid economic growth, the disease once associated with poverty is becoming a chronic illness [2]. Economic level directly influences the incidence of shigellosis [3]. In China, shigellosis incidence dropped by 46.29% from 2005 to 2010, but the disease burden was not evenly distributed [4]. Although the water and sanitation infrastructure has improved in China, the usage of safe water and proper sanitation varies widely across the country [5]. From 2006 to 2011, Wuhan City reported 36 487 bacillary dysentery cases, with a mean annual incidence of 77.4 per 100 000 population (ranging from 68.9 to 81.5 per 100 000) [6]. Furthermore, the reported incidence varies in Anhui Province, Sichuan Province and Baise City [3, 7, 8]. A relatively high disease burden was found in Wuhan City compared to Chinese average level [9, 10].

Kermack and McKendrick [14] built a Susceptible–Infectious–Recovered (SIR) and a Susceptible–Infectious–Susceptible (SIS) model, on which the Susceptible–Infectious–Recovered–Water (SIRW) model is based. The SIRW model is used to examine disease outbreaks caused by waterborne pathogens [11]. The mathematical model named Susceptible–Exposed–Symptomatic/Asymptomatic–Recovered–Water/Food (SEIARW) is suitable to explore the mechanism of water/food-born disease, such as the transmission process of a small-scale outbreak in a school in Changsha City [12]. Thereafter, a study reported the application of the SEIARW model to the Hubei Province and confirmed that it is mainly concentrated on person-to-person transmission [13]. However, its prediction of transmissibility was at the entire province level. The study also showed that the incidence in the province was heterogeneous. Among its 13 cities or prefectures, Wuhan City had the highest incidence [17]. Therefore, the transmission characteristics in Wuhan City might be different from the rest of the province. It is essential to employ the SEIARW model to calculate the transmissibility in a large city, such as Wuhan City. Previous studies always applied the basic reproduction number (*R*_0_) to evaluate infectious diseases [14–17]. Because *R*_0_ is difficult to quantify, here, the effective reproduction number (*R*_eff_) was adopted to evaluate the shigellosis transmission instead [18].

Recently, studies have suggested a correlation between shigellosis incidence and climatic factors, including atmospheric pressure, sun hours, temperature, relative humidity, wind speed and precipitation [6, 19–22]; however, they have not clarified how meteorological factors affect the transmission of the disease. Climate may directly influence the survival of shigellosis in the environment, or indirectly affect the transmission of shigellosis through human behaviours [23]. However, there is still an epidemiological ‘black box’ [24] (a system of which the input and the output are known but of which nothing is known about intermediate steps) between the climatic factors and the incidence of the disease. It is essential to study the association between shigellosis transmissibility and meteorological variables. That might supply us with a new pathway to explore the transmission mechanism of the disease.

The SEIARW dynamics model was used to simulate shigellosis transmission to further quantify the transmissibility and estimate its correlation with meteorological factors.

## Methods

### Source of data

Data regarding shigellosis from January 2005 to December 2017 were obtained from Wuhan City through the China Information System for Disease Control and Prevention (CISDCP). Daily meteorological data (average daily value of atmospheric pressure, temperature, relative humidity, precipitation, wind speed and sun hours) from 2008 to 2017 were obtained from the National Population Health Data Centre (https://www.ncmi.cn) and the demographic data including total population, birth rate and death rate were obtained from the Wuhan Statistical Yearbook.

### Model development

**Fig. 1.**
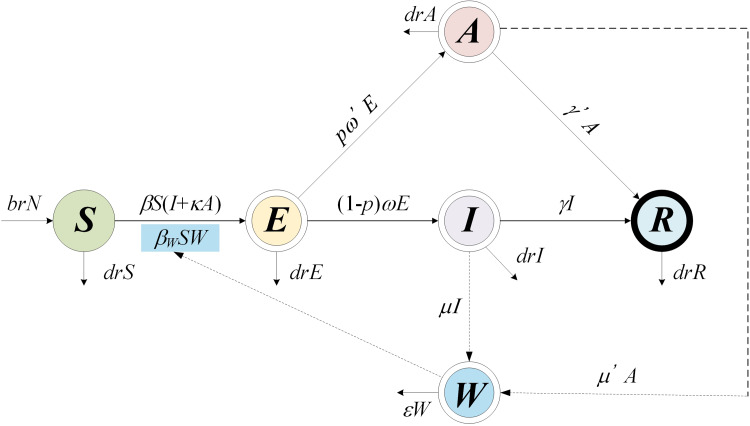
Flow diagram of SEIARW model of shigellosis.

Based on our previous study [12], the population in the person-to-person route differed into five compartments, and another reservoir route including water and food was also simulated in the model (Fig. 1). As data for the infectious disease model covered more than a decade, we added demographic data such as birth and death rates. The definition and units of measurement of the storage places are shown in Table 1. Model assumptions are listed as follows:

- Shigellosis is not vertically transmitted, and newly born individuals are all susceptible. The natural change of population was considered in the model, with the natural birth rate denoted by *br*, and the death rate by *dr*.
- Susceptible people may be infected after contact with contaminated water/food or symptomatic/asymptomatic infected people. The infection rate coefficients are *β_W_* and *β*, respectively.
- The average incubation period of illness is 1/*ω*. We assumed the transition rate from *E* to *I* and *E* to *A* is equal. The asymptomatic proportion was denoted by *p* (0 ⩽ *p* ⩽ 1). Therefore, the transferred rate from exposed to asymptomatic and symptomatic is denoted by *pωE* and (1 – *p*)*ωE*, respectively.
- People in *I* or *A* will be recovered after a symptomatic infectious period (1/*γ*) or an asymptomatic infectious period (1/*γ'*), respectively.
- Symptomatic and asymptomatic individuals can shed the *shigella*, with a *μI* and *μ'A* shedding rate, respectively.
- *Shigella* spp. die in water/food after a period, and the daily rate of decrease of the pathogen is *ɛW*.

**Table 1.** Description and unit of variables in SEIARW model

Variable	Description	Unit
*S*	Susceptible individuals	Individuals
*E*	Exposed individuals	Individuals
*I*	Symptomatic individuals	Individuals
*A*	Asymptomatic individuals	Individuals⋅
*R*	Recovered individuals	Individuals
*W*	Pathogen concentration in water/food reservoir	Cells/ml
*N*	Total number in population	Individuals

The differential equations of the model are as follows:
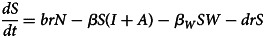

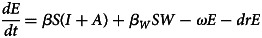

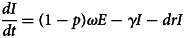

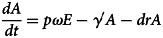

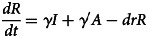

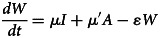
Twelve parameters were used to develop the SEIARW model, including *β*, *β_W_*, κ, *p*, *ω*, *γ'*, *γ*, *c*, *ɛ*, *μ*, *μ'*, *br* and *dr*, which were defined and valued in Table 2. Let *N* denotes total number in population, we perform substitution *b* = *βN*, *b_W_* = *μβ_W_N/ɛ*, *s* = *S/N*, *e* = *E/N*, *a* = *A/N*, *i* = *I/N*, *w* = *ɛW/μN*, *r* = *R/N* and *μ* = c*μ'*, and use the dimensionless differential equations of the model (Model 1):
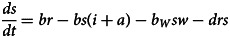

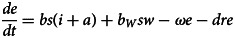

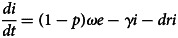

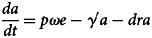

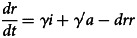

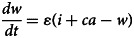
The person-to-person route of shigellosis transmission is described by the following equations (Model 2):
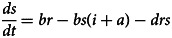

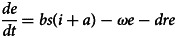

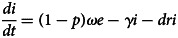

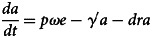

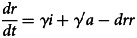
We calculated the *R*_eff_ to estimate transmissibility by the value of *b* simulated for each time segment such as seven parts in 2014 (Fig. 2). The equation is as follows:
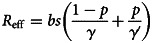

**Fig. 2.**
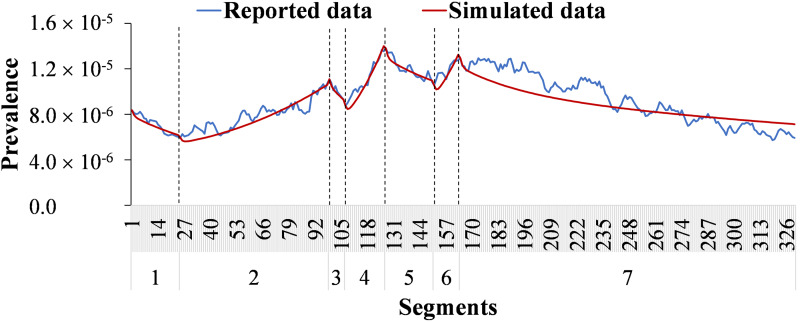
The division segments according to the prevalence reported per day of Wuhan City in 2014.

**Table 2.** Descriptive information of parameters in SEIARW model

Parameter	Description	Unit	Value	Range	Method
*β*	Transmission relative rate from person-to-person	/individuals/days	－	⩾0	－
*b*	Scaled transmission relative rate from person-to-person	/days	－	⩾0	Curve fitting
*β_W_*	Transmission relative rate from water/food-to-person	ml/cells/days	－	⩾0	－
*b_W_*	A product of two transmission rates divided by pathogen lifetime relative rate	/days	－	⩾0	Curve fitting
κ	Relative transmissibility of asymptomatic to symptomatic individuals	1	0.3125	0–1	Reference [12, 13, 25]
*p*	Proportion of the asymptomatic individuals	1	0.1	0.0037–0.27	Reference [12, 13, 25]
*ω*	Incubation relative rate	/days	1	⩾0	Reference [12, 13, 25]
*γ*	Recovery rate of the symptomatic individuals	/days	0.0741	⩾0	Reference [12, 13, 25]
*γ'*	Recovery rate of the asymptomatic individuals	/days	0.0286	⩾0	Reference [12, 13, 25]
*ɛ*	Pathogen lifetime relative rate	/days	0.6931	0–1	Reference [12, 13]
*μ*	Person-to-reservoir contact rate ‘shedding’ by symptomatic individuals	cells/ml/(symptomatic individuals)/day	－	－	－
*μ'*	Person-to-reservoir contact rate ‘shedding’ by asymptomatic individuals	cells/ml/(asymptomatic individuals)/day	－	－	－
*c*	Shedding rate of the asymptomatic compared with the symptomatic individuals	1	0.3125	0–1	Reference [12, 13]
*br*	Birth rate of the population	1	0.000017	－	Wuhan Statistical Yearbook
*dr*	Death rate of the population	1	0.00012	－	Wuhan Statistical Yearbook

－ means not applicable.

### Estimation of parameters

We set *κ*, *ω*, *p*, *γ*, *γ'*, *c* and *ɛ* as 0.3125 (0–1), 1.0000 (0.3333–1), 0.1000 (0.0037–0.27), 0.0741 (0.0477–0.1428), 0.0286 (0–0.0357), 0.3125 (0–1) and 0.6931 (0–1), respectively, based on previous research (Table 2) [12, 13, 25]. According to the Wuhan City Yearbook, the annual birth rate and death rate are 0.0063 and 0.0045, respectively. We set *br* and *dr* per day as 0.000017 and 0.000012, respectively. The total population from 2005 to 2017 in Wuhan City is shown in Supplementary Table S1. Parameters *b* and *b_W_* were generated by calibration of Models 1 (SEIARW) and 2 (SEIAR) to previously reported shigellosis data.

### Simulation method and statistical analysis

To evaluate the contribution of the two transmission routes, we employed a method called ‘knock-out’ simulation whose theory comes from the technique of gene ‘knock-out’, which could make one of an organism's genes inoperative. Furthermore, we performed four scenarios with the ‘knock-out’ simulation (cutting off diverse routes): (a) *b* and *b_W_* were set to 0; (b) *b* was set to 0; (c) *b_W_* was set to 0; and (d) *b* and *b_W_* were taken value from the model fitting (None). The *b_W_* = 0 means either *μ* = 0 or *β_W_* = 0 or both are 0 so this is not just setting water/food-to-person contact rate to 0. The total number of infected cases was monitored in each scenario.

We adopted the decreasing the infectious period (DIP) method to estimate the intervention effectiveness in 2017 of Wuhan City. The baseline in 2017 was *R*_eff_ = 1.16, *γ* = 0.0741 and infectious period (IP) 13.5 days. The DIP was assumed as follows: (1) an infected individual would go to a hospital immediately after becoming symptomatic, and (2) the hospital can diagnose and treat the patient. We calculated the value of *R*_eff_, IP and DIP with a change proportion (Δ*γ*) of 10% and 20%, respectively. The DIP was calculated as follows:
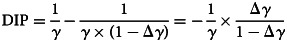


Berkeley Madonna 8.3.18 (Robert Macey and George Oster of the University of California at Berkeley. Copyright ©1993–2001 Robert I. Macey & George F. Oster) was adopted for simulations. Calibration was performed via minimizing the root-mean-square deviation and the Runge–Kutta method of order four with tolerance set to 0.001 was adopted to solve the differential equations [12−14, 23, 24]. Microsoft Office Excel 2019 (Microsoft, Redmond, WA, USA) was employed to run the data analysis, develop figures and calculate the value of *χ*^2^ to evaluate the goodness of fit of the SEIARW model.

Data in 2014 were divided into seven parts that were called segments and the time step size of the simulation was set to be one day (Fig. 2). We summarised the 3 months with the highest number of new cases each year to estimate the seasonality of illness onset and used the peak *R*_eff_ to estimate the seasonality of transmission.

The Spearman's correlation coefficient (*r*_s_), determined using SPSS 21.0 (IBM Corp., Armonk, NY, USA), was used to evaluate how meteorological variables were related to shigellosis. To consider the lag effect between risk factors and disease, in one study of meteorological variables and shigellosis, it was found to be 3 and 4 days [20]. We lagged the meteorological variables by 1, 2, 3 and 4 days and calculated Spearman's correlation coefficient for each lag. Furthermore, we matched the daily meteorological variables with the *R*_eff_ of the corresponding period, then calculated the mean of the meteorological variables and estimated the correlation.

### Sensitivity analysis

We performed a sensitivity analysis for seven parameters which were divided into 1000 values based on their ranges from reference, then the standard deviation (s.d.) and mean of the simulation were obtained. The sensitivity analysis was only performed for data in 2014 to avoid the yearly repeated simulation. Usually, the transmissibility of infectious diseases is different between rising and reduction trends. The year 2014 was divided into seven segments and each was calibrated (Fig. 2).

## Results

### Epidemiology description of shigellosis in Wuhan City

**Fig. 3.**
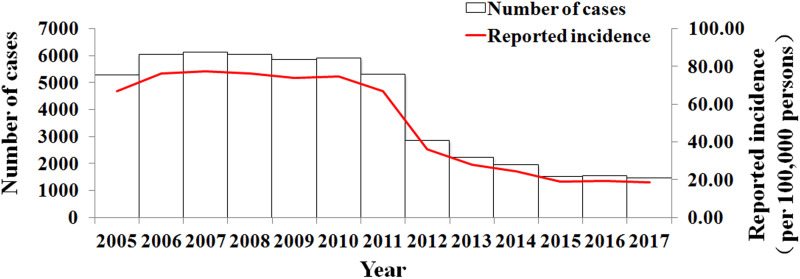
Number of reported cases and incidence rate of shigellosis from 2005 to 2017 in Wuhan City, China.

As shown in Figure 3, Wuhan City reported a total of 51 948 shigellosis cases from 2005 to 2017 with a range of yearly incidence rate from 18.44 to 77.53 per 100 000 persons (median: 66.79 per 100 000 persons). The incidence rate and the number of reported cases had a significantly decreasing trend (test for linear trend *χ*^2^ = 9.735, *p* = 0.002). From 2005 to 2011, the incidence rate and the number of reported cases were stable. However, from 2011 to 2015, the incidence rate and the number of reported cases decreased quickly, followed by a steady trend after 2015.

### Curve fitting and estimation of transmissibility

**Fig. 4.**
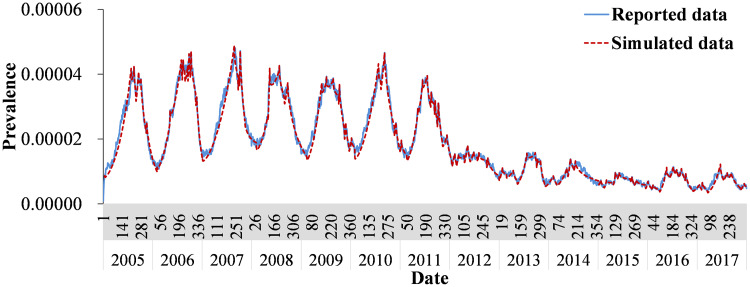
Fitting effectiveness between SEIARW model and reported data in Wuhan City, China from 2005 to 2017.

In Figure 4, Model 1 fitted the data well (*χ*^2^ = 0.00046, *p* > 0.999). The *b_W_* was 3.4331 × 10^–10^ (95% CI 1.4179 × 10^–11^–6.7245 × 10^–10^) and *b* was 0.0892 (95% CI 0.0741–0.1044). The values of *b* and *b_W_* with no obvious seasonality in different segments per year are shown in Supplementary Table S1.

**Fig. 5.**
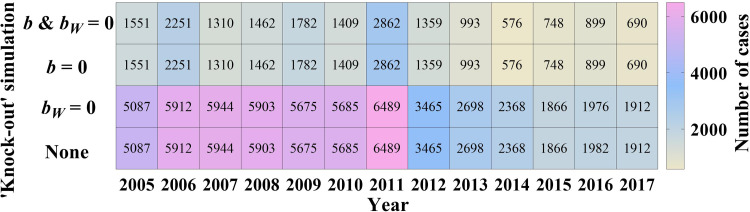
Simulated number of cases by four scenarios (*b* and *b_W_* = 0; *b* = 0; *b_W_* = 0; and *b* and *b_W_* ≠ 0 defined as ‘None’).

We found the person-to-person transmission route is more significant (Fig. 5) according to the ‘knock-out’ simulation. We obtained the same results when cutting off the route from person-to-person (*b* = 0) as cutting off double routes (*b* = 0 and *b_W_* = 0). Meanwhile, a consistent number of cases was observed between interrupting water/food-to-person route (*b_W_* = 0) and control (*b* and *b_W_* ≠ 0).

**Fig. 6.**
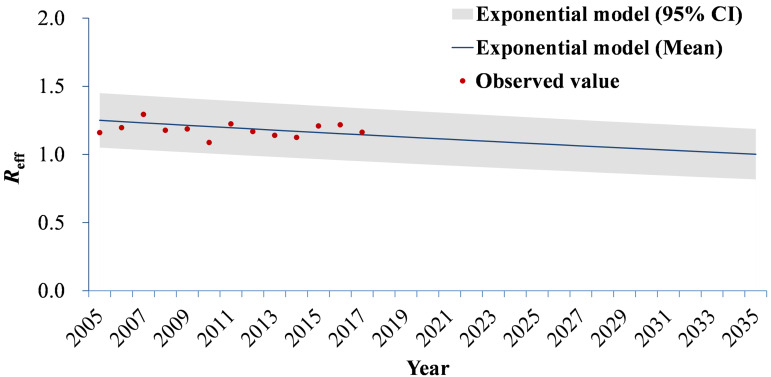
Simulated trends of *R*_eff_ in Wuhan City, China.

Although *R*_eff_ had a relative fluctuation from 2005 to 2017, the trend was gradually decreasing. The mean values (Table 3) were calculated from 1.09 (95% CI 0.96–1.22) to 1.29 (95% CI 0.94–1.65). Figure 6 shows that an exponential model fits the trend of *R*_eff_ well (*χ*^2^ = 0.03473, *p* > 0.999). It was forecasted to reach the epidemic threshold of 1.00 (95% CI 0.82–1.19) in the year 2035, according to the exponential model.

**Table 3.** The yearly *R*_eff_ *was* calculated by the SEIAR model from 2005 to 2017 in Wuhan City, China

Year	Mean	Lower bound of 95% CI	Upper bound of 95% CI
2005	1.16	0.91	1.41
2006	1.20	1.05	1.34
2007	1.29	0.94	1.65
2008	1.18	0.95	1.40
2009	1.19	1.02	1.36
2010	1.09	0.96	1.22
2011	1.23	1.04	1.41
2012	1.17	1.04	1.29
2013	1.14	0.94	1.34
2014	1.12	0.93	1.32
2015	1.21	1.06	1.35
2016	1.22	1.00	1.44
2017	1.16	0.90	1.43

**Fig. 7.**
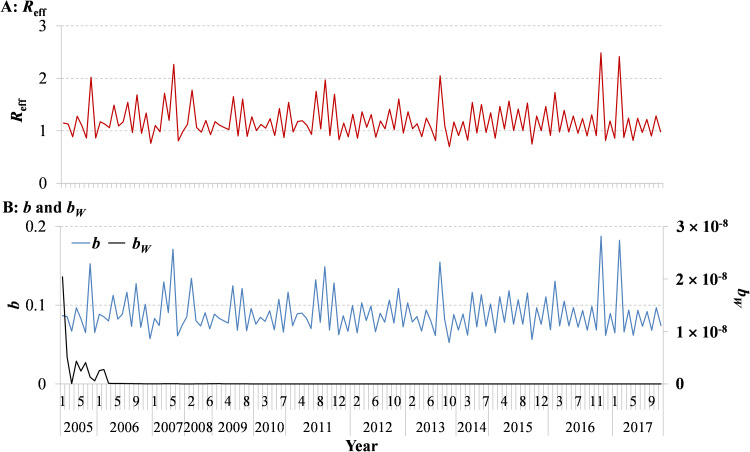
Simulated trends of *R*_eff_, *b* and *b_W_* per segment from 2005 to 2017 in Wuhan City, China. (A) *R*_eff_; (B) *b* and *b_W_*. The numbers above the Years represent segments per year. The detailed dates of several numbers above each year are shown in Supplementary Table S1.

**Fig. 8.**
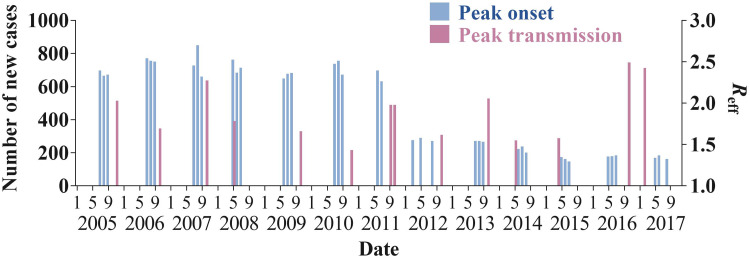
The peak of shigellosis onset (3 months of the top number of cases) and transmission (*R*_eff_).

We observed that the transmissibility of shigellosis fluctuated within the range of 1–2 (Fig. 7A). The parameter *b* had the same trend as *R*_eff_. Meanwhile, we found that the contribution of water/food-to-person had some effects in the previous 2 years (Fig. 7B), but the transmission from water/food had been interrupted after 2006. In addition, the results showed that there was obvious seasonality of illness onset, mainly concentrated from June to September (Fig. 8). The seasonality of peak transmissibility (*R*_eff_) was mainly concentrated in October.

### Correlation between shigellosis and meteorological factors

**Fig. 9.**
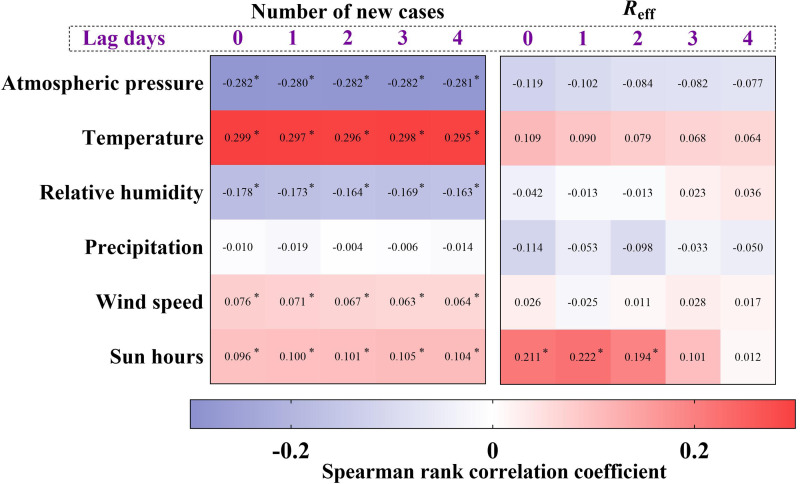
The correlation between meteorological variables with new cases and *R*_eff_. *Significant difference from 0.

Our results (Fig. 9) showed significant correlation between new cases and atmospheric pressure (*r*_s_ = −0.282), temperature (*r*_s_ = 0.299), relative humidity (*r*_s_ = −0.178), wind speed (*r*_s_ = −0.076) and sun hours (*r*_s_ = 0.096); there was no significant correlation between new cases and precipitation (*r*_s_ = −0.010). However, we just found a positive significant correlation between *R*_eff_ and sun hours (*r*_s_ = 0.211). Furthermore, there was no obvious change in *r*_s_ when the meteorological variables were lagged by 1, 2, 3 and 4 days.

### Modelling DIP

We further simulated different scenarios to decrease the infectious period (increase the percentage of parameter *γ*) and to determine the conditions to achieve *R*_eff_ = 1.00. The results showed that the transmission of shigellosis would probably be interrupted (*R*_eff_ = 0.99) if the infectious period were shortened by 2.25 days (Table 4).

**Table 4.** Reducing the *R*_eff_ down to the transmission threshold (*R*_eff_ = 1.00) by shortening the symptomatic infectious period

Δ*γ*	*R*_eff_	Infectious period (days)	Decreasing the infectious period (days)
0%	1.16	13.50	0.00
10%	1.07	12.27	1.23
20%	0.99	11.25	2.25

### Sensitivity analysis

**Fig. 10.**
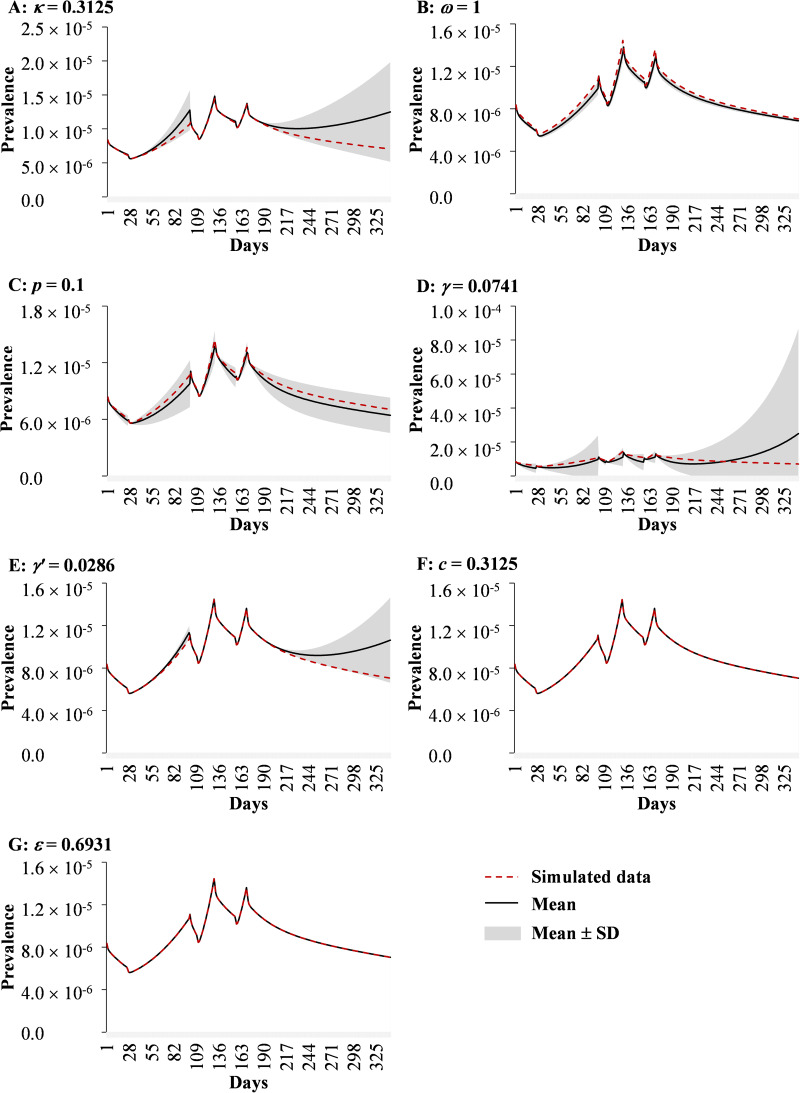
The sensitivity analysis of parameters in the SEIARW model in 2014. Red curve, black curve and grey shaded area represent the parameters used in our model, mean value and range of standard deviation, respectively. (The upper limit of the shaded area represents the most extreme possibility when taking the maximum value of the parameter.) A narrow grey shaded interval means less sensitivity to this parameter.

We obtained the same results in the simulation when setting the parameters *c* and *ɛ* (Fig. 10G) to the maximum, minimum and mean values, respectively. The SEIARW model was not sensitive to three parameters, including *ω*, *ɛ* and *c*, but it was sensitive to four parameters, including *κ*, *p*, *γ* and *γ'* (Fig. 10).

## Discussion

This study innovatively explored the association between meteorological variables and *R*_eff_ of shigellosis. We investigated the transmissibility and forecasted the transmission of shigellosis by using the SEIARW model for the whole population in a large city and further explored the relationship between transmissibility and meteorological factors.

### Simulation validity

This study adopted the SEIARW dynamics model to explore the feature of shigellosis transmission. Although this transmission model has a disadvantage in that it could fit the data poorly when the disease is sporadic, shigellosis was epidemic in Wuhan City during the study period. Therefore, our model is suitable to perform the simulation.

We added the birth and death rates in the assumptions to the model. Compared with a previous shigellosis model of Hubei Province, we further considered the variation of demographic features [13]. According to the *χ*^2^ test, the SEIARW model fits the reported data well. Our findings suggest the validity of the simulation, and this is consistent with our previous findings [12, 13, 25]. Our results indicate that the simulation was more sensitive to parameters *κ*, *p*, *γ* and *γ'*. We recommend that further study could collect the parameters *κ*, *p*, *γ* and *γ'* from first-hand data, rather than derived from references.

### Description of shigellosis

Several studies indicated a reduction of the incidence of shigellosis in China [2, 4, 9, 26, 27], but the disease has remained a high burden in Wuhan City. A study indicated the incidence heterogeneity in Hubei Province [13]. Compared with Hubei Province overall, the incidence in Wuhan City was the highest (median: 66.79 per 100 000 persons). Kotloff *et al*. indicated that water/food transmission may still play a significant role in transmitting the disease, which is often further propagated by person-to-person transmission [1]. This is consistent with our result of ‘knock-out’ simulations when setting *b_W_* = 0, which means either *μ* = 0 or *β_W_* = 0 or both are 0. This might relate to the following reasons: cleaner water and toilets reducing the frequency of people coming into contact with water polluted by *Shigella* spp. (it can be considered that *β_W_* = 0); or managing shigellosis cases so that they rarely excrete pathogens to drinking water and food (it can be considered that *μ* = 0). Although transmission has been interrupted when *b* and *b_W_* are set to zero, there is still a section of exposed people in compartment E who will develop cases. The SEIAR model across sex was employed in a study to discuss interpersonal transmissibility; different transmissibility was found in different genders [25]. Meanwhile, a study of Hubei Province confirmed the interruption of the water/food-to-person route [13], which is closely related to the improvement of water, lavatories and food safety in China. Therefore, the control of shigellosis should be focused on person-to-person transmission, including interventions of case isolation, treating the patients and hand washing.

### Transmissibility and seasonality of shigellosis

Compared with influenza, Ebola virus disease and norovirus infection, shigellosis is not highly contagious [14–16, 28]. We found the range of *R*_eff_ to be 1.09–1.29 in Wuhan City (higher than the epidemic threshold of 1.00). This finding suggests that one symptomatic/asymptomatic individual can infect at least 1.03 susceptible persons.

According to an exponential model of the transmissibility of shigellosis, *R*_eff_ has a decreasing trend. We forecast that shigellosis in Wuhan City would not lose epidemic transmission capability until 2035 (*R*_eff_ = 1.00). On one hand, meteorological factors can influence the transmissibility of shigellosis [4]. On the other hand, our disease control strategy may be strengthened, which might also affect *R*_eff_. However, we found that the transmission of shigellosis would be interrupted in Hubei Province after 2029 [13]. This may be related to the fact that Wuhan City has the highest incidence of shigellosis in Hubei Province. Also, the transmission features in different areas may vary, and a relatively high disease burden may increase the duration of the transmission. The transmissibility in different areas may be influenced by environmental factors, economic factors, education and population density.

Simulated scenarios to shorten the infectious period showed that reducing the infectious period by 2.25 days achieved *R*_eff_ = 0.99. Therefore, reducing the infectious period is especially important for secondary prevention. We should take measures such as isolation or antibiotic treatment as early as possible.

The obvious seasonality of shigellosis onset was observed in this study (mainly concentrated from June to September). This may be related to the summer vacation of primary and middle school students from July to August. Furthermore, we found that there is a seasonality of peak transmissibility (*R*_eff_), which was mainly concentrated in October. We consider that it may be related to the outbreak of shigellosis in October, and the seasonality of transmissibility needs to be further studied.

### Correlation between shigellosis and meteorological factors

Several studies have demonstrated the correlation between reported cases of shigellosis and climatic factors over many years, such as temperature (positive), rainfall (positive), relative humidity (positive), wind speed (negative), sunshine duration (negative) and atmospheric pressure (negative) [20, 23, 29, 30]. In this study, we found a significant correlation between new cases and atmospheric pressure, temperature, wind speed and sun hours. A previous study indicated a positive correlation between the monthly incidence of shigellosis and both average temperature (*r*_s_ = 0.878) and average rainfall (*r*_s_ = 0.931) in Beijing City [31]. However, we did not find a significant correlation between new cases and precipitation (*r*_s_ = 0.010); and we found a weak correlation between new cases and temperature (*r*_s_ = 0.299). These findings suggest that analysing the correlation with daily data might yield weaker results than with monthly data [22]. A previous study showed a weak negative correlation of sun hours with shigellosis incidence in Chaoyang City of the Liaoning Province (load factor: −0.15) [32]. In our study, a weak positive correlation was found between new cases and sun hours. This might be because the sun hours affected human behaviours such as increasing travelling.

*R*_eff_ is an indicator reflecting person-to-person transmissibility. Two previous studies found that person-to-person is an important mode of transmission [13, 25], as did our study. We consider that meteorological factors mainly influence the survival of bacteria and human behaviour. Rainfall and high temperatures can enhance the reproductive capacity of bacteria. Social behaviours such as shaking hands, embracing and kissing can increase the risk of infection. Although the attribution analysis could not be directly employed between disease and meteorological variables, we calculated Spearman's correlation coefficient between meteorological variables and *R*_eff_. We found a weak positive correlation between *R*_eff_ and sun hours. This is different from the results of the correlation between meteorological variables and new cases. Some studies have found no correlation or a small negative correlation between shigellosis cases and sun hours [31, 33]. Sun hours may influence human behaviour. For example, it may reduce social behaviours such as shaking hands if sweating. This variation in results also may be due to the nonlinear relation between *R*_eff_ and new cases. The incidence of shigellosis could be related to meteorological and other variables.

### Limitations

Some studies have indicated an effect of meteorological variables on shigellosis incidence with a lag of 3 or 4 days or even several weeks [20, 34]. As the incubation period is typically 1–3 days [35], a lag effect of more than 4 days was not analysed in this study. In addition, seasonality should be considered in future studies and incorporated in model development. Tien and Earn collected the key data for the SIWR model, including the parameters *α* (person–reservoir contact rate (‘shedding’) and 1/*ξ* (pathogen lifetime in water reservoir)), which is very important for modelling results [15]. In this study, the key parameters *ɛ* and *c* of our model were from a reference, not first-hand data, which might underestimate the contribution of water/food in shigellosis transmission. We could not consider the population shifting in the compartmental model because we did not collect the internal migrants. Furthermore, further analysis such as Poisson regression should be performed to explore the association between shigellosis onset and several additional factors (economic, population shifting, age and so on). For further work that the pathogen concentration in the water is measured on a regular basis, be considered and the incidence of shigellosis could be related to this and other variables.

### Conclusions

In Wuhan City, the incidence of shigellosis was relatively high from 2005 to 2017. The main transmission pattern is person-to-person. The transmission of shigellosis might be stopped in the year 2035 according to our forecast. Meteorological factors, especially daily atmospheric pressure and temperature, may influence the epidemic of shigellosis.

## Data Availability

The datasets used and analysed during the current study are available from Qi Chen (chenqi8700@qq.com) on reasonable request.

## Supplementary material

For supplementary material accompanying this paper visit http://dx.doi.org/10.1017/S0950268821002363. click here to view supplementary material
