# Supplementary figures and images for: Transmission pattern of shigellosis in Wuhan City, China: a modelling study
Source: Epidemiol Infect. 2021 Nov 2;149:e249. doi: 10.1017/S0950268821002363 (PMC8697311; doi:10.1017/S0950268821002363)

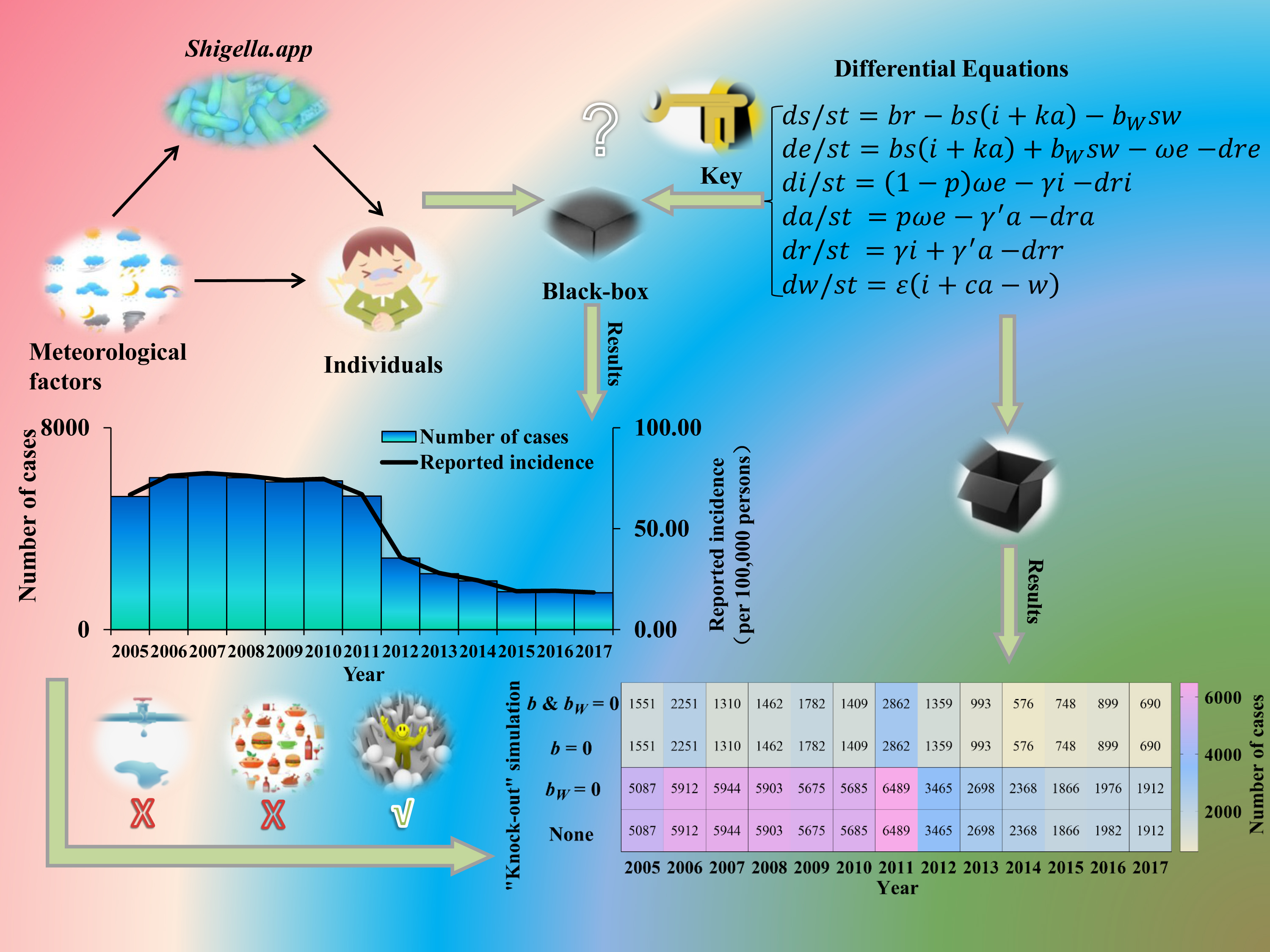

Supplement: Supplementary file 1 [file hygsup.zip › S0950268821002363sup002.jpg]
